# Inside the Clockwork of the ECHO Factorial Trial: A Conceptual Model With Proposed Mediators for Prevention of Emotional Problems in Children

**DOI:** 10.3389/fpsyg.2021.703224

**Published:** 2021-06-21

**Authors:** Jo Magne Ingul, Kristin Martinsen, Frode Adolfsen, Anne Mari Sund, Kristin Ytreland, Elisabeth Valmyr Bania, Carina Lisøy, Lene-Mari Potulski Rasmussen, Ida Mari Haug, Joshua Patras, Linda M. Collins, Philip C. Kendall, Simon Peter Neumer

**Affiliations:** ^1^Regional Centre for Child and Youth Mental Health and Child Welfare (RKBU), Department of Mental Health, Faculty of Medicine and Health Sciences, NTNU - Norwegian University of Science and Technology, Trondheim, Norway; ^2^Department of Psychology, Faculty of Social Sciences, University of Oslo, Oslo, Norway; ^3^Center for Child and Adolescent Mental Health, Eastern and Southern Norway, Oslo, Norway; ^4^Faculty of Health Sciences, Regional Centre for Child and Youth Mental Health and Child Welfare North, UiT The Arctic University of Norway, Tromsø, Norway; ^5^St Olav's University Hospital, Trondheim, Norway; ^6^Department of Social and Behavioral Sciences, College of Global Public Health, New York University, New York, NY, United States; ^7^Department of Psychology, Temple University, Philadelphia, PA, United States

**Keywords:** child emotional problems, prevention, cognitive behavior therapy, evidence-based interventions, emotion, optimization, factorial design, mediators

## Abstract

Having interventions that are not only evidence-based and effective but also cost-effective and efficient is important for the prevention and treatment of child and adolescent emotional problems. A randomized clinical trial (RCT) tests the total interventions effect but does not address specific components of the intervention. In this article the hypothesis and a conceptual model of the ECHO study are presented and discussed. The ECHO intervention consists of three different components each containing two levels of intervention. By using a cluster randomized factorial design, children aged 8–12 at 40 schools across Norway will be randomized to eight different experimental conditions investigating the optimal balance between effect, cost-effectiveness, and efficiency. The article presents the design and the different components being tested and discusses how optimalization can be reached through this innovative design. The article also discusses how interventions can be improved by investigating and understanding the mechanisms of change within psychological interventions. For each of the three components in the study we consider the mediators that could be active within the intervention and how the study investigates such mediation. The results will contribute to a better understanding of how psychological interventions work and how we intend to optimize the EMOTION intervention.

## Introduction

Preventing the development of emotional disorders in children is important for the individual and society. The global burden of disease study indicates a growth of these problems in the general population (Vos et al., 2016). Providing the most effective interventions while spending as little time and effort as possible is the ideal. To achieve this, we need to optimize evidence-based strategies by better understanding the mechanisms of change in psychological interventions (Kazdin, 2007). Optimization has been defined as; “the process of identifying an intervention that provides the best expected outcome obtainable within key constraints imposed by the need for efficiency, economy, and/or scalability” (Collins, 2018, p. 12). The first part of this article presents the conceptual model of the ongoing ECHO study and how a factorial design is used to examine and optimize an already evidence-based intervention (Martinsen et al., 2019) through experimentally testing 3 components. The second part of the article discusses how the different components are hypothesized to work, and how the interventions and their components possibly mediate change (Kazdin, 2007). The objective of the article is to describe how a novel and innovative design is being used to optimize an evidence-based intervention, and how the different components and mechanisms of the study are based on theoretical considerations.

Emotional problems in the form of anxiety and depression are common in young people, with a lifetime prevalence until the young person is 18 of 11.7% for depression and 31.9% for anxiety (Merikangas et al., 2010). Anxiety and depression are associated with significant impairment (Kendall et al., 2010; Rohde et al., 2013; Swan and Philip, 2016), and many children are at risk for poor outcomes and future mental health problems if left untreated (Cummings et al., 2014). Unfortunately, studies of service use show that many children go untreated and are often not referred for help before functional impairment and comorbidity has increased substantially (Merikangas et al., 2011; Sund et al., 2011). In depression, anxiety is the most common comorbid disorder, with estimates of comorbidity ranging between 15 and 75%, whereas in anxiety, depression generally seems to be less comorbid ranging from 10 to 15% (Angold et al., 1999; Costello et al., 2003).

There have been different explanations for this co-occurrence. For instance, the tripartite model (Clark and Watson, 1991) and the multiple pathways model (Cummings et al., 2014) have tried to explain the different aspects of anxiety and depression and their co-occurrence, but as of yet there is not one empirical model that can account for both unique and common factors. However, the focus on co-occurrence and common factors, and common psychological processes in these disorders have led to the development of transdiagnostic intervention models. Transdiagnostic models are especially well-suited for general mental health and preventive settings (Clark, 2009). The aim of prevention efforts is to reduce the risk for disorders by reducing incidence, prevalence and recurrence (Muñoz et al., 1996). Given the high prevalence of mental disorders in the population such efforts are imperative to reduce the burden for the individual, their families, and to society at large (WHO, 2004). Preventive efforts aim to reduce risk factors and enhance protective factors associated with the identified problem. It is common to distinguish between universal, selective and indicated prevention (Haggerty and Mrazek, 1994). In universal prevention the whole population is targeted. Selective and indicated intervention are targeted approaches where the children are recruited based on a common risk factor, or a heightened symptom level, respectively (Haggerty and Mrazek, 1994; Greenberg, 2010).

Examining all types of prevention programs in a meta-analysis, Werner-Seidler et al. (2017) reported small, but positive effects on anxious and depressive symptoms for interventions in a school setting compared to a control group [for depression Effect Size (ES) = 0.23, and for anxiety ES = 0.20]. Stockings et al. (2016) reported similar results, internalizing symptoms were reduced by preventive interventions, but with lower effect size for universal interventions (ES = 0.15), than for selective interventions (ES = 0.20). Mychailyszyn et al. (2012) examined school-based interventions in particular, also reporting positive effects for anxious and depressed children with moderate effect sizes for interventions targeting anxiety (ES = 0.32 for universal interventions and ES = 0.79 for indicated interventions), and small effect sizes for interventions targeting depression (ES = 0.20). Hence, while some studies report larger symptom reductions for indicated and selective interventions compared to universal interventions (e.g., Calear and Christensen, 2010; Teubert and Pinquart, 2011; Mychailyszyn et al., 2012), the results are mixed.

## Optimization in a Factorial Design

The ECHO study addresses the moderate and at times conflicting results and is aimed at optimizing an evidence-based indicated, preventive intervention for children with emotional problems. Most interventions comprise multiple components that work together to produce change. A traditional randomized controlled trial (RCT) is useful to identify whether total interventions are effective, but such a design does not answer which intervention components produce change. The ECHO study uses a cluster randomized full factorial design, with three components, each with two levels. This design gives eight different combinations (see Table 1) of the components that participating children at 40 schools across Norway will be randomized to. The study plans to include 796 children aged 8–12 years scoring one standard deviation or more over the mean on primary outcome measures of anxiety and or depression to the intervention (see https://clinicaltrials.gov/ct2/show/NCT04263558?term=Neumer&draw=2&rank=2 for details). Thus far over 400 children have been included. A factorial experiment is the ideal design for answering questions about the effect of different components and whether the different components impact the effect of another. The presence vs. absence of each component is manipulated as an independent variable and corresponds to a factor in an experimental design (Collins et al., 2014). The components are (1) group intervention/DIGGI: Here the CBT based EMOTION intervention (Martinsen et al., 2017) consisting of 16 group sessions will be tested against a partially digital EMOTION intervention (DIGGI), designed for this study consisting of the same 16 sessions but where 8 sessions are given face to face and 8 sessions are digital (see Table 2 for details). Here the child completes the different sessions at home on a Pad or PC. In addition, Virtual Reality (VR) technology will be used during behavioral experiments (see Table 2) in both versions of the intervention. Here, 360° videos of challenging tasks and situations, using head mounted displays, are used to train and expose the children; (2) High/low parental involvement: In this component the effect of parental participation is tested. Parents will be randomized to either a 5-session parent group focusing on how to help an anxious or sad child (high involvement) or they will be randomized to a condition where they receive a brochure with psychoeducational information for parents (low involvement; see Table 2 for details); and (3) Measurement feedback/no measurement feedback: The third component will test the effect of a Measurement Feedback System (MFS). Here half the participants will be randomized to a condition where the children use an MFS app to answer questions about their development weekly, while the other half is randomized to a condition where this app will not be used.

**Table 1:** Components and experimental conditions in the ECHO study.

	**Component**		
**Experimental condition**	**1 Emotion vs. DIGGI**	**2 Parental involvement**	**3 Measurement feedback system (MFS)**
1	Emotion	High	Yes
2	Emotion	High	No
3	Emotion	Low	Yes
4	Emotion	Low	No
5	DIGGI	High	Yes
6	DIGGI	High	No
7	DIGGI	Low	Yes
8	DIGGI	Low	No

**Table 2:** Content of EMOTION; child and parent sessions.

	**Content of child sessions**	**Delivery format**		**Content of parent sessions**	
		**Group version (16 group sessions)**	**DIGGI (8 group + 8 digital sessions)**	Parental sessions (5 group sessions)^*^	
1	Introduction/establishing rules	Group	Group		
2	House of change/conceptual model	Group	DIGGI		
3	Recognizing feeling, setting goals	Group	Group	Motivation/goalsetting^*^Facilitate parent-child relationship	1
4	Emotion focused coping	Group	DIGGI		
5	Problem solving	Group	Group		
6	Thoughts influences feelings	Group	DIGGI		
	Problem solving in real situations	(not in group version)	Group	Positive parenting and reinforcement^*^	2
7	Problem solving applied to anxiety	Group	DIGGI		
8	Cognitive change/Behavioral experiments	Group VR	Group VR		
9	Cognitive change/Behavioral experiments Positive self-concept	Group	DIGGI		
10	Cognitive change/Behavioral experiments Positive self-concept	Group VR	Group VR	House of change/behavioral experiments^*^Educate parents in recognition of emotions	3
11	Cognitive change/Behavioral experiments Positive self-concept	Group	DIGGI		
12	Cognitive change/Behavioral experiments Positive self-concept	Group	DIGGI		
13	Cognitive change/Behavioral experiments Positive self-concept	Group VR	Group VR	Cognitive restructuring/behavioral experiments^*^Parental engagement in problem solving	4
14	Integrating knowledge Behavioral experiments Positive self-concept	Group	DIGGI		
15	Integration of coping skills Behavioral experiments	Group	(not in DIGGI version)		
16	Closing up	Group	Group	Closing up^*^Experiencing parental modeling behavior	5

* *Parents and child together*.

### Data Collection

The ECHO study uses multi-method measurement: Children aged 8–12 years old complete self-report questionnaires. Parents report on children's symptoms, user satisfaction and their own symptoms. Teachers report on child symptoms and academic achievement. Group leaders in the intervention and head of the municipal services involved report on their user satisfaction and attitudes toward evidence-based interventions. EMOTION outcomes are also multimethod, including children, group leaders and service leaders participating in the study. The primary outcomes are changes in depressive and/or anxious symptoms using the Multidimensional Anxiety Scale for Children (MASC; March et al., 1997) and the short version of the Mood and Feelings Questionnaire (SMFQ; Angold et al., 1995) to measure change (Neumer et al., 2021). Secondary outcomes are also collected [for details see the protocol paper (Neumer et al., 2021)]. The main aim of the ECHO study is to optimize the intervention and provide knowledge about the contribution of each component to the outcome of the intervention (main effects) and to detect possible interaction effects between the combinations of the components.

## The Conceptual Model of the ECHO-Study

**Figure 1:**
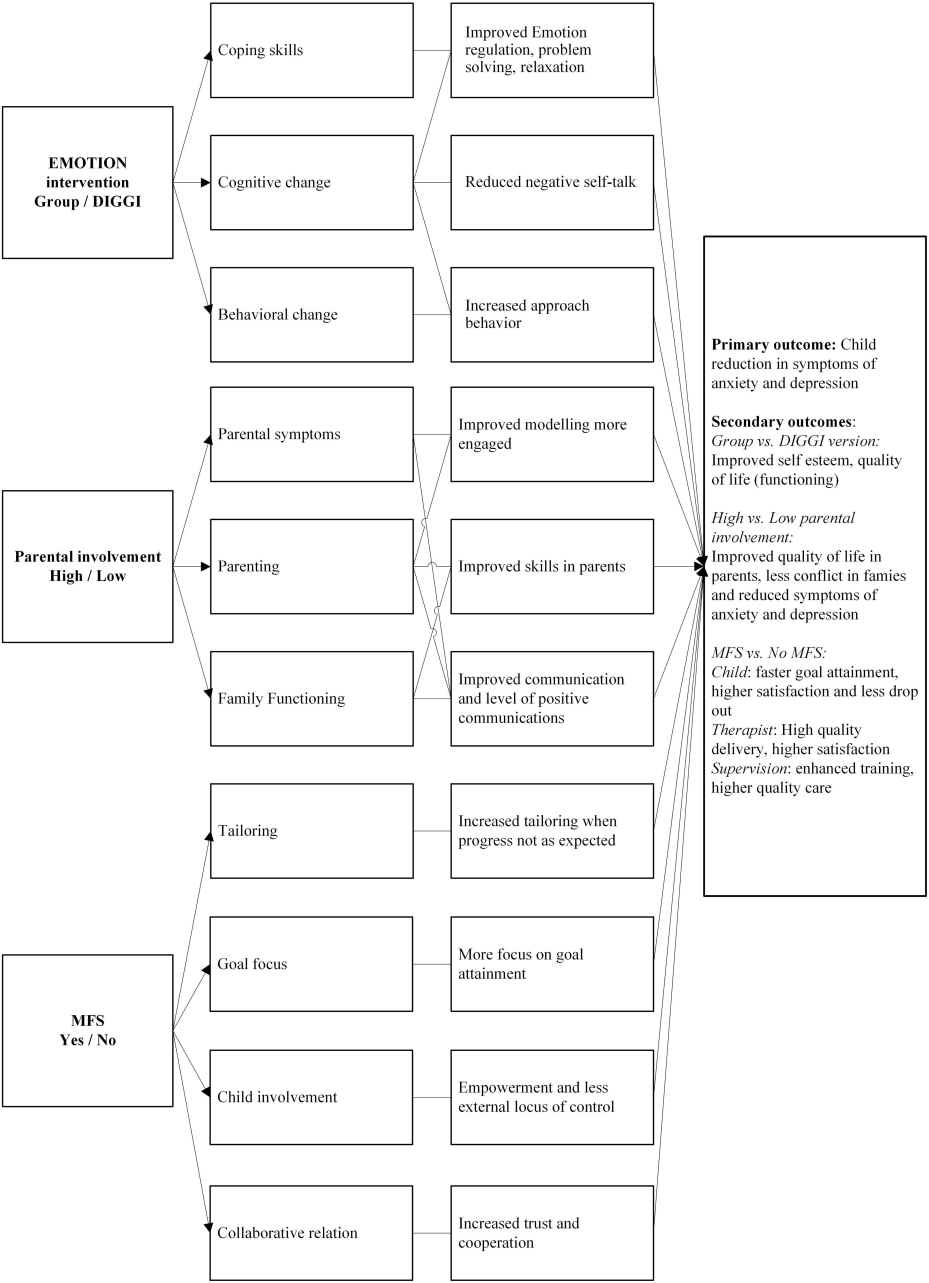
The conceptual model of the ECHO study. The model depicts how different components in the study will impact mediators and combine to produce change in primary and secondary outcomes.

The conceptual model of the ECHO study is presented in Figure 1. The model is based on a developmental view of how psychopathology or symptoms develop (Rutter and Sroufe, 2000), how cognitive behavioral therapy (CBT) conceptualizes change, and research of risk factors and mediators of change in CBT for youth anxiety and depression. According to the developmental psychopathology perspective multiple factors contribute to maladaptive (and adaptive) outcomes in any person, and these factors and their contribution to any development will vary in every individual, meaning that there will be many different pathways to any developmental outcome (Cicchetti and Rogosch, 1996). Hence, the development of psychopathology is the result of the balance between risk and protective factors present in any child's life at any given time. These factors interact to produce an outcome that may be changed through influencing any one of the contributing factors. For the ECHO study, the intervention is directed at changeable risk factors and mediators of child symptoms of anxiety and/or depression and is intended to influence the balance between the risk and protective factors.

CBT (Kendall, 2011) predicts that through improving emotion regulation (ER), altering behavioral patterns of avoidance and passivity, and changing maladaptive thinking patterns and attitudes, youth will experience change in symptoms of anxiety and depression. It is hypothesized that these mechanisms will operate regardless of the format of delivery, as the partially digital (DIGGI) version of the EMOTION-intervention is designed to teach the same psychoeducational, cognitive and behavioral skills to the children as the group version. It is assumed that both in the full original format and in the DIGGI version, the same mediators will operate to produce change.

The conceptual model also posits that components related to parents and parenting are modifiable through an intervention. Due to mixed findings in research regarding the effect of parental involvement on child symptoms (Silverman et al., 2008) we hypothesize that there will be no difference between the two conditions (high vs. low parental involvement) with respect to the primary outcome.

Regarding the third component (MFS vs. no MFS) the model hypothesizes that routine feedback to service providers might mediate the result of the intervention. In ECHO, the use of MFS will increase systematic user feedback that does not depend on individual service providers. Based on earlier meta-analysis and research we expect an enhanced outcome through process feedback independent of the intervention delivered. As shown in Figure 1, we hypothesize that using MFS vs. not using MFS could mediate outcome at different levels. For children, we anticipate they are more actively involved in their own progress by using the feedback system, this can lead to learning coping skills quicker and hence improve outcomes. Monitoring the children's goals and symptom levels during the intervention also provides for opportunities for the group leaders to tailor the intervention, and this may lead to better outcomes in the MFS-condition.

The primary aim of the ECHO study is to learn how best to optimize the intervention by providing knowledge about the contribution of each component to the outcome of the intervention (main effects) and to detect possible interaction effects between the combinations of the components. This results in the following primary hypotheses related to the components:

1. There will be no differences in outcomes between the EMOTION intervention and DIGGI.
2. There will be no differences in outcomes between high and low parental involvement.
3. Using MFS will result in better outcomes compared to not using MFS.
4. Hence, the study predicts that the most optimized version of the study will be using the DIGGI version for children, with low parental involvement, but MFS being used by children and group-leaders will show superior outcome results compared to other combinations of components.

## Intervention Components of ECHO

### Component 1: EMOTION Intervention vs. DIGGI

The EMOTION intervention (Norwegian version: Martinsen et al., 2017, US version: Kendall et al., 2013) is transdiagnostic and was developed based on disorder specific protocols for treating anxiety and depression in children (Kendall and Hedtke, 2006; Stark et al., 2007). The co-occurring nature of anxiety and depression in children (Avenevoli et al., 2001; Cummings et al., 2014) and the many similar components of disorder-specific interventions was some of the background for this transdiagnostic approach. The objective was to develop an intervention flexible enough to target both problems using strategies targeting transdiagnostic mechanisms of change. These mechanisms were disturbances in cognitive processing, coping skills, problem-solving and behavioral strategies (Kendall et al., 2014).

The original version of the EMOTION manual was structured with 20 child group sessions (children meeting twice a week for 10 weeks) and seven parent meetings (children attending three of these). Children learn new skills in the first half of the intervention, in the second half of the intervention they practice these skills and focus on cognitive restructuring and behavioral experiments. Enhancing the children's self-esteem is also a major focus in the last part of the intervention.

In a randomized controlled study examining the effectiveness of the EMOTION, children in the intervention group reported significant reductions in symptoms of anxiety and depression, with the intervention group reporting almost twice the reduction in symptoms as the control group (Martinsen et al., 2019). Group leaders running the intervention in schools and managers in the first line services reported that the intervention was useful for the children targeted, but also that there was need for more flexibility in the intervention and that time constraints running the program was a challenge (Rasmussen et al., 2020).

Based on the results and feedback from providers, EMOTION was revised adding more flexibility (Martinsen et al., 2017). The current version used in the ECHO study consists of 16 child sessions and 5 parental sessions. Due to the implementation barrier that time constraints represent a search for alternative methods of delivering the intervention was sought. Recently internet-based interventions targeting anxiety and depression in young people have reported positive effects (Richardson et al., 2010). A meta-analysis (Hollis et al., 2017) found moderate ES for internet-based interventions targeting depression (ES = 0.16–0.62), and high effect sizes comparable with what is found in individual settings for anxiety (ES = 0.53–1.41). In a study by Richardson et al. (2010) young people aged 7–25 reported positive reductions in anxious and depressive symptoms. They were also satisfied with the internet delivered interventions, but other studies have indicated possible high attrition rates for purely internet delivered interventions (Waller and Gilbody, 2009; Vangberg, 2013). With this background a partially digital version (DIGGI) was developed to be examined in the ECHO study. Here, the 16 sessions were split into 8 sessions provided in groups, and 8 digital sessions for the child to complete at home on a Pad or PC. The digital sessions were created using Articulate Storyline 3 (2019) and published as internet-based sessions using GitHub. For the purpose of this study the sessions were placed in the learning management system Canvas, to which group participants were invited. The DIGGI version is interactive, requires little writing- and reading skills and has a playful approach to learning about emotions, cognitions, and behavioral strategies (see Table 2 for an overview).

### Component 2: Parental Involvement

The Parental sessions of the EMOTION (Martinsen and Keeping, 2017) were developed based on parental involvement strategies from evidence-based interventions targeting anxiety and depression (Kendall and Hedtke, 2006; Stark et al., 2007). CBT that involves parents in child therapy, has in a recent meta-analysis been found to be a well-established treatment for anxious children (Higa-McMillan et al., 2016). However, studies show mixed results as to whether parental involvement adds to the effect of child-alone anxiety treatment (Breinholst et al., 2012; Brendel and Maynard, 2014). It is possible that studies without added effect have failed to address parental and familial factors impacting childhood anxiety (Banneyer et al., 2018). A review of parental involvement in childhood anxiety treatment by Barmish and Kendall (2005) suggest that the way, and the degree to which parents are involved varies a lot between studies, and that conclusions about effects are difficult to draw (Manassis et al., 2014).

Interventions for depression have shown poorer results for younger children than adolescents (Cuijpers et al., 2020). Parental involvement in treating children for depression typically involves facilitating the routines of the child's daily life (e.g., sleep, nutrition, and activities). Although recommendations for improving effectiveness of CBT therapy for childhood depression has included parent training (Weisz et al., 2006) the causal role of parental involvement is still uncertain (Mcleod et al., 2007). Also, what parts of CBT that are most important for depressed children are not clarified and needs to be explored (Asarnow et al., 2002). A few studies have included parents, both in clinical and high-risk samples of children. In some studies, the involvement of parents does not add to effectiveness (Brent et al., 1997; Clarke et al., 1999; Stikkelbroek et al., 2020), and a recent meta-analysis did not identify parental intervention as a moderator (Eckshtain et al., 2020), whereas this was not supported by another meta-analysis by Oud et al. (2019). The mixed findings lead to a question about how parental interventions can be improved.

Originally, the parental involvement of EMOTION consisted of 7 meetings, 3 of which were together with the child (Martinsen and Keeping, 2017). Based on results from a previous study (Martinsen et al., 2019), the parental component was reduced to 5 sessions with parents where children still attend 3 of these (Martinsen and Keeping, 2017). Parents receive psychoeducation, learn about positive parenting, behavioral experiments, problem solving and cognitive restructuring (see Table 2 for details).

### Component 3: Measurement Feedback

The third component comprises the use of an MFS vs. no such feedback. The use of feedback systems where providers receive feedback routinely on participants progress is a promising intervention in therapeutic contexts with adults and children (Gondek et al., 2016; Tam and Ronan, 2017; Bergman et al., 2018). MFS has in adult studies shown to increase effect of psychosocial interventions, including reduction in symptoms (Gondek et al., 2016), faster improvement (Bickman et al., 2011), less drop-out (Lambert et al., 2018), and it seems to be more effective for so-called not-on-track patients (Gondek et al., 2016). The effect of using MFS is less researched among children and young people in psychosocial interventions, though it seems to have positive, but small effect sizes of 0.20–0.32 (Tam and Ronan, 2017).

Several MFS have been developed (Lyon et al., 2016) and a few tested for children and adolescents, for example, the Treatment Response Assessment for Children (TRAC; Cheron et al., 2019), and Contextualized Feedback system (Bickman et al., 2011), yet there are some challenges to implementation. Among them are high costs, few validated instruments for children and adolescents are included, data safety and privacy regulations are not met, and systems are inflexible with little opportunity for adaptation (e.g., choosing instruments) (Lyon et al., 2016).

The MFS developed for the present study is called MittEcho (Norwegian, MyEcho translated), and consists of the MittEcho app and the MittEcho publication portal. The main function of the MittEcho app is collecting children's data during an intervention. In the early stage of the intervention, the children identify up to three personal, idiographic aims. Each week thereafter, they are asked to evaluate their progress on each aim. In addition, the children complete a short measure of depression and anxiety symptoms in the app based on the Behavior and Feelings Survey (Weisz et al., 2019). The MittEcho publication portal graphically displays the results from the weekly questions and the participants' personal goals. Here group leaders monitor and follow the development of each participant and adjust the intervention to fit the individual's needs.

## Optimization Through Implementation Assessment and Systems Intervention

Implementation support is important for the success of evidence-based practices (EBP). Several multi-level frameworks have been developed to identify key factors in implementation work and to identify how they interact to facilitate or inhibit program effectiveness (e.g., Damschroder et al., 2009; Fixsen et al., 2009; Aarons et al., 2011).

The context you implement in is often complex and difficult to describe and will therefore have an impact on the implementation process in general (May et al., 2016). Structural, organizational, innovation, provider and child factors are combined and important for the intervention outcome, but also the implementation outcome. Proctor et al. (2011) define implementation outcomes as “the effects of deliberate and purposive actions to implement new treatments, practices, and services” (Proctor et al., 2011, p. 65). This can be assessed through different outcomes, e.g., adoption, satisfaction, fidelity, cost, penetration, and sustainability (Proctor et al., 2011; Chaudoir et al., 2013).

Within the study design and the context of intervention delivery (i.e., first line services) in ECHO, different experimental conditions will have an impact on the different implementation strategies (e.g., training, use of measurement feedback system), which in turn could affect different implementation outcomes such as adoption and fidelity (Proctor et al., 2011). In this study, the 8 different conditions convey a meaningful way of evaluating different implementation strategies. The DIGGI version of EMOTION reduces the number of in-session meetings where group leaders need to be present. Thus, the main goal of the digitalization is to decrease group leaders' workload, which again can increase feasibility and acceptability of the intervention. Improved implementation outcomes (e.g., feasibility, acceptability) have a positive impact on delivery within the services, which ultimately have a positive effect on the children (Proctor et al., 2011). If the digital version of the program shows similar results on children's symptom level as the full version, the use of DIGGI can potentially reach more children by freeing up resources for the services.

As with DIGGI, the low parental involvement condition could potentially be less resource demanding for the services, as less presence from the group leaders is required. Evaluating the low vs. high parental involvement will not only provide insight regarding mechanism of change for children's symptomology, but also offer some results regarding implementation outcomes, such as feasibility with the program for group leaders. On the other hand, low parental involvement might require extra effort by the group leaders in keeping the children engaged, and completing all tasks (e.g., homework, DIGGI sessions, and/or using the app), which could potentially reduce the usefulness of the intervention for the children.

The last component being evaluated, using MFS or not, could also be viewed in an implementation framework. For group leaders, the MFS provide an opportunity to tailor the intervention, thereby optimizing the intervention outcome for these children. In an implementation context, enhanced tailoring of the intervention should produce less attrition and relapse. Thus, in a long-term perspective, outcomes would improve for more children, and resources in the services could be saved.

## Mediators and Mechanisms in Optimization

Although research in prevention and psychotherapy has moved forward, there is still a lack of knowledge about how and why different interventions work. To be able to optimize any treatment or preventive effort we need to identify the components that account for change in outcomes. Which components work? And for whom? Such an understanding would enable us to pinpoint the important components of the treatment that must not be changed or diluted (Kazdin, 2007). In the next section optimization is described through processes that may account for change in outcomes. This often implies studying mediators and mechanisms. Mediation analysis is a statistical strategy used to study hypothesized indirect effect pathways, in which the intervention affects mediators, which in turn influence the distal outcome significantly (Mackinnon and Luecken, 2008). There are several requirements for establishing a variable as a mediator: association, specificity, consistency, timeline and gradient (Kazdin, 2007). Mediators do not necessarily describe the precise process of change, but they may point to processes that lead to change. The term mechanism is therefore often used to describe the process(es) or event(s) that have been tested and to be responsible for change and is a more specific explanation of why an independent variable has effect on the dependent variable.

## Mediators Related to the Components in the ECHO Study

### Component 1: Processes Responsible for Treatment Gains in the EMOTION Intervention

**Figure 2:**
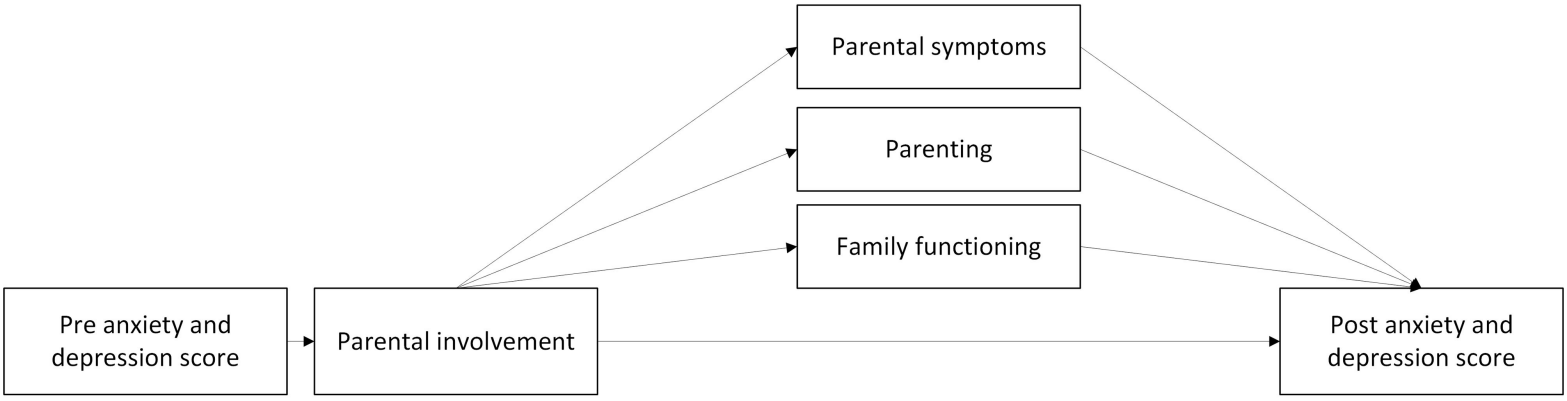
Proposed mediators of change with the EMOTION component.

A defining feature of transdiagnostic treatments is that they aim to treat multiple problems using a common set of techniques, targeting a set of core underlying processes (Kendall et al., 2014). While the study of transdiagnostic mechanisms is still nascent, research efforts to identify core mechanisms of change for anxiety and depression has made some progress. Several potential mediators have been identified, although further investigation is required to demonstrate formal mediation or to establish possible causal relationships associated between mechanisms and outcomes (Chu and Harrison, 2007). In the following section, we present candidate transdiagnostic mediators that may impact what we propose are transdiagnostic mechanisms (see Figure 2).

#### Impact Through Behavioral Change

Avoidance appears to be a central feature and a maintaining factor in both anxiety and depression (Chu et al., 2014). Avoidant behavior is negatively reinforced by escaping a stressor, and the escape also prevents opportunities for positive reinforcement (Jacobson et al., 2001). Thus, exposure strategies aim to reduce avoidance in anxious children through a gradual approach to feared situations while employing coping skills or relaxation. Results from a meta-analysis (Chu and Harrison, 2007) suggest that CBT for anxious youth is particularly effective for targeting behavioral outcomes, consistent with theories of anxiety that prioritize exposure and activation of fear networks in producing greater approach behaviors (Craske and Mystkowski, 2006). There is also a consistent relationship between depressed mood and avoidant processes, such as decreased activity, social withdrawal, and isolation (Chu et al., 2014). Children with both depression and anxiety are more likely to use avoidant plans than non-anxious/non-depressed children (Dickson and Macleod, 2004). Children with depressive symptoms also have difficulties in generating approach plans and approach goals. Kovacs and Yaroslavsky (2014) have found that children at risk for depression are vulnerable to the changing contexts of daily life, having difficulties managing their own sadness (mood repair) leading to passivity. For sad children, behavioral strategies therefore typically focus on pleasant activity scheduling and behavioral activation and frequent rewards to break their cycle of withdrawal and passivity.

#### Cognitive Change as Mediator—The Way We Think Affect the Way We Feel

The cognitive model (Beck, 1967) proposes that inaccurate beliefs and maladaptive information processing (repetitive negative thinking) cause and maintain depression. It also suggests that when information processing is corrected, symptoms of depression are reduced. Negative styles of thinking such as pessimistic or hopeless explanatory styles predicts depression (Lakdawalla et al., 2007) and interventions that modify pessimistic explanatory style and other negative thinking styles have effects on depression (e.g., Horowitz and Garber, 2006). Furthermore, some studies have found initial evidence suggesting that improvement in explanatory style mediates intervention effect on depressive symptoms in children (Yu and Seligman, 2002; Brunwasser et al., 2018). In depression, a reduction in cognitive negative thoughts, especially perfectionism, has been found to be an important mediator among depressed adolescents (Stice et al., 2010).

Negative self-talk (negative automatic thoughts) also appears to maintain anxiety (Kendall et al., 2014). Children with anxiety and mood disorders report more dysfunctional and negative beliefs than other children (Beck, 2005). Anxious children also report more negative self-talk than non-anxious children, and anxious youth report more negative than positive self-talk (Sood and Kendall, 2007). Furthermore, anxious youth are more likely to interpret ambiguous situations as threatening and respond with avoidance strategies (Barrett et al., 1996). Indeed, negative self-talk is most often anticipatory and future-oriented and involves perceived threats which are often exaggerated (Kendall et al., 2014). Self-talk in depression, often denoted rumination, is on the other hand more often past-oriented and involves loss, feelings of worthlessness or hopelessness following events.

Transdiagnostic treatments for anxiety and depression aim to reduce negative self-talk and achieve a healthier ratio between negative and positive self-talk. Cognitive change may be achieved through both cognitive and behavioral strategies, targeting different cognitions in anxiety and depression. The two most common misappraisals in emotional disorders are overestimating the likelihood of a negative event and catastrophizing the consequences of such an event (Moses and Barlow, 2006). Such faulty threat appraisals then lead to avoidance, which maintains the disorder. CBT seeks to enhance threat reappraisal both through exposure and cognitive restructuring techniques. In the U.S., a large trial found that the introduction of (a) cognitive restructuring and (b) exposure tasks accelerated the rate of progress on symptom severity (Peris et al., 2015). However, improvement in anxious self-talk was not a significant mediator of treatment gains (Kendall et al., 2016).

#### Enhancing Coping Skills as Mediator

The ability to *cope* with stressful events and circumstances and *regulate emotions* across situations may also play a primary role in psychopathology in children. Coping and emotion regulation skills therefore play a central role in transdiagnostic models of preventive interventions. The skills are related to processes such as emotional understanding, problem solving, relaxations skills and skills to handle difficult situations (Chu and Harrison, 2007). Findings from a previous trial where the effects of EMOTION was investigated, revealed a negative association between children's symptoms of anxiety and depression and emotion regulation (Loevaas et al., 2018). This was consistent with findings from Compas et al. (2017) meta-analysis of 212 studies. Results indicated that adaptive coping and emotion regulation was associated with lower symptoms of externalizing and internalizing problems in children and adolescents. Prins and Ollendick (2003) reviewed the evidence for cognitive and coping variables as mediators of CBT for anxious youth. They found few studies testing mediation, but many studies had assessed pre- to post-treatment outcomes of cognitive or coping process. They found greater change in cognition and coping-related measures following CBT when compared with wait-list-conditions. Kendall et al. (2016) identified that coping efficacy (reported ability to manage anxiety provoking situations) temporally precede and mediate treatment gains for anxiety in youth, indicating that improvements in coping efficacy is an important mediator of change. According to Kendall et al. (2016), exposure may facilitate the development of coping skills moving from passive to active strategies and the use of physiological, and cognitive strategies such as relaxation and problem solving.

### Component 2: Improved Modeling, Skills, and Communication in Parents May Help Improve Child Symptoms

**Figure 3:**
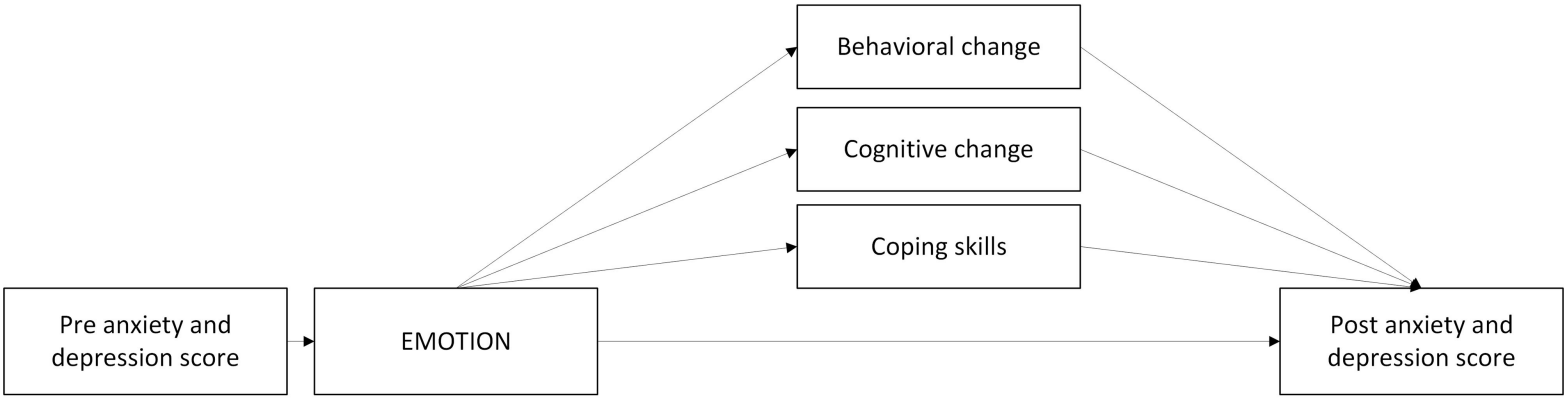
Proposed mediators of change with the parental involvement component.

Parents are involved in children's development in many ways, and risk factors for negative development and maintenance of child emotional problems have been linked to parents, their behavior and the environment they provide for their child. In this section, possible mediators of childhood anxiety and depression associated with parents, measured in the ECHO study, are presented and discussed (see Figure 3).

#### Parental Anxiety and Depression

Parental internalizing (disorders and) problems are associated with corresponding problems in their offspring (Cooper et al., 2006; Colletti et al., 2010). Several studies have found a relationship between parental anxiety and anxiety in their child (Last et al., 1991; Cooper et al., 2006; Johnson et al., 2006). Colletti et al. (2010) also suggests a link between parent-child depressive symptoms and parent-child anxious symptoms. In a study of anxious 8–12-year-olds, paternal rejection, and anxious and depressive symptoms in fathers were associated with less favorable child (Liber et al., 2008). The authors suggested that parenting style of these fathers were more rejective due to a depressed mood. An examination of several theory-driven intervention mediators revealed that reductions in modeling of anxiety and global parental distress, measured by the Brief Symptom Inventory (Derogatis et al., 1974), which includes symptoms of anxiety and depression, were significant mediators for child anxiety (Ginsburg et al., 2015).

#### Parenting

Parenting is the sum of a parent's interaction with their child and is associated with child anxiety and/or depression, through for example parental control (Soenens et al., 2008), modeling behavior (Breinholst et al., 2012) and parent-child relationship (Brumariu and Kerns, 2010; Wu and Lee, 2020). *Parental control* is defined as overprotection, excessive regulations on the child's activities, decision-making and imposing on how the child should think and feel (Wood et al., 2003). Casline et al. (2018) found that parental use of force and punishment when their child responds to situations with fear and/or avoidance is associated with higher levels of child anxiety symptoms. Parental control can affect the child's locus of control, lead to children perceiving events as out of their control and reduce their self-competence. Both cognitive styles (locus of control and self-competence) are related to internalizing problems (Chorpita and Barlow, 1998; Affrunti and Ginsburg, 2012). Soenens et al. (2008) found a link between perceived psychological control and youth depressive symptoms over time.

Children may learn anxious behaviors by seeing parents model them, and through reinforcement or accommodation of such practices (Wood et al., 2003; Ginsburg et al., 2015). By observing their parents, children may adopt unhealthy strategies (Barrett et al., 1996), which can lead to maladaptive problem-solving strategies (Ugueto et al., 2014). In a group of children of anxious parents, Ginsburg et al. (2015) found parental anxious modeling behavior to be a significant mediator for child anxiety.

The specific *parent-child relationship* may serve as a protective factor for internalizing symptoms. As measured by self-report, parent-child relationships at the age of nine affects the trajectories of internalizing symptoms, i.e., anxiety and depression, up to the age of 18 (Wu and Lee, 2020). Insecure maternal attachment is associated with anxiety and depression in the offspring (Brumariu and Kerns, 2010). In early adolescence, lower parental attachment scores, i.e., low communication, low trust and high alienation, predicted an increase in depressive symptoms one year after (Sund and Wichstrøm, 2002).

These findings suggest potential mediators, and that targeting specific parenting behaviors and lowering parents' overall distress levels may be important in reducing child anxiety symptoms (Ginsburg et al., 2015). Studies on depression indicate that improving relationships in the family, teaching parents to praise the child, reducing criticism, engaging the child in fun activities, and increasing family time has a positive effect on child depressive symptoms (Duong et al., 2016; Moreno-Peral et al., 2020). The content of and how to implement these interventions warrants further research.

#### Family Functioning

Family functioning is multifaceted and can be broadly defined as the way family members behave toward each other, i.e., conflicts, relationships, and overall functioning in the family (Bögels and Brechman-Toussaint, 2006). Worse family functioning, as perceived by the child, correlated with child depression and anxiety diagnosis, compared to a control group (Stark et al., 1990). Dysfunction in the family is associated with diminished treatment response for children with obsessive-compulsive disorder and anxiety disorders (Peris et al., 2012; Schleider et al., 2015). Fosco et al. (2016) examined family conflict as a mechanism of change and found that family conflict increased between 6th and 9th grade for both control and intervention groups, but the slope was steeper in the control group. They also found that more family conflict predicted an increase in youth depressive symptoms in the same period. Trudeau et al. (2016) found that relationship problems mediated symptoms of depression (ES = 0.25) 11 years after an intervention. In a depression prevention program for youth, Duong et al. (2016) found that parent-child communication, as measured by child self-report on caregivers' openness to communication and parent-child communication about problems and feelings was a mediator for child outcome.

### Component 3: Regular Feedback and Child Goal Orientation Enables Tailoring and Better Individual Help (MFS)

**Figure 4:**
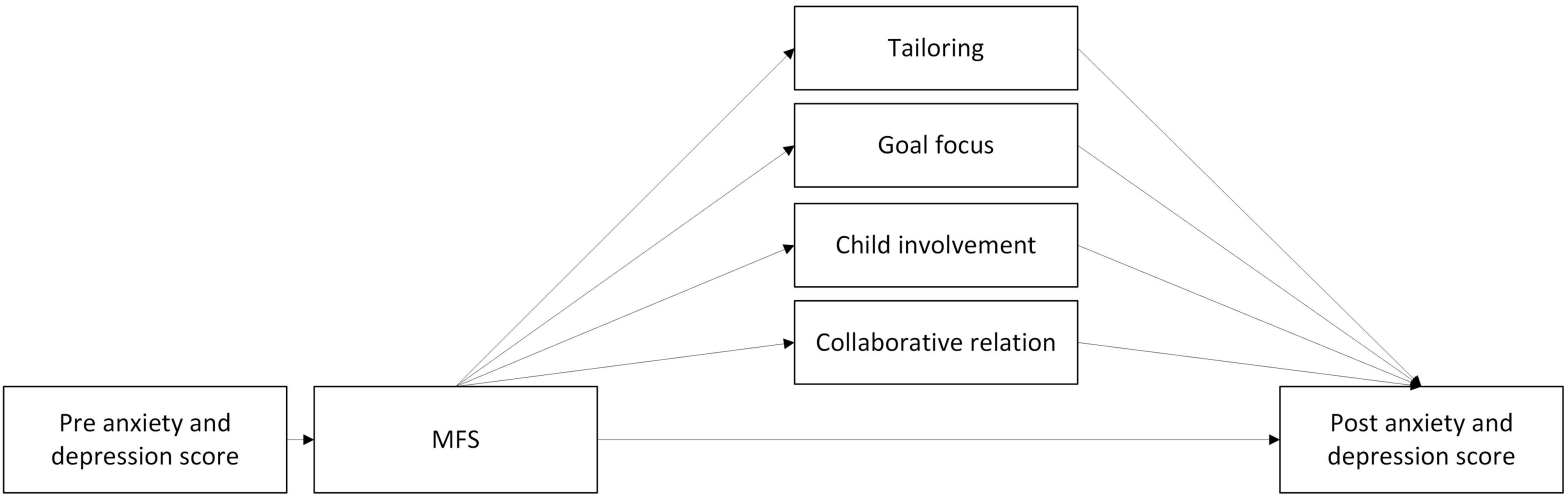
Proposed mediators of change with the measurement feedback system component.

A Cochrane report for MFS with children and adolescents (Bergman et al., 2018) concluded that there are promising aspects in using MFS to optimize interventions, although results thus far are inconsistent. New results concerning the application of MFS for adolescents in Norway (Tollefsen et al., 2020a) support this view. Though most of the research on child psycho-social interventions and use of MFS has been done in clinical contexts and individual therapy (Tam and Ronan, 2017; Bergman et al., 2018), the same principles could apply in a group-based preventive intervention such as EMOTION. There are two main differences between the context of the ECHO study and previous research; (1) group leaders track up to seven children at once, whereas in individual therapy they would only track one child. (2) Opportunity to tailor the intervention may be limited due to the manual-based nature of the EMOTION. Though meditators and mechanisms of change with the use of MFS are not yet empirically established, research from other populations and contexts, as well as theory, may help to identify candidates (see Figure 4).

#### Tailoring

Repeated data collections by MFS provides service providers with regular feedback about clients' progress during the intervention. In the context of the ECHO study, MFS can be used to tailor the intervention to the needs of children and to assess intervention outcomes at short-term intervals. MFS provides the group leader with systematic information on the children's symptom burden and goal progress, and the group leader's actions on this information may be the primary mechanism through which MFS works.

The Contextualized Feedback Intervention Theory states that among the firsts steps toward changing practice for therapists is acknowledging that current status is discrepant from the desired status (Riemer et al., 2005). It has been demonstrated that therapists are overly optimistic when evaluating client's progress, and not accurate in predicting treatment failure (Lambert, 2010), which in turn will prevent them from changing the treatment plan. Feedback from MFS provide information on the current status and may contribute to earlier detection if progress is not as expected. This makes it possible to tailor interventions to individual needs sooner (Douglas et al., 2015; Bergman et al., 2018).

MFS also seems to have a gradient relation to treatment outcome, in line with Kazdins (Kazdin, 2007) description of mediators (Bickman et al., 2011, 2016). Bickman et al. (2016) found a dose-response relation between how often therapist received feedback, and outcomes in patients in a youth outpatient clinic.

#### Goal Focus

Most of the current MFS use nomothetic measures (i.e., questionnaires with predefined concepts). However, these may not capture topics that are of most importance and interest to children (Lloyd et al., 2019). Idiographic measures, such as personal aims chosen and evaluated by children themself, can support child progress. A meta-analysis of working with goals in psychosocial interventions showed small, but positive, effects (d = 0.34) (Epton et al., 2017). Results from Tollefsen et al. (2020a) indicated that MFS can support the service provider by promoting co-operation with the adolescents helping them to focus on their personal aims for the intervention. MFS has the potential to support children and adolescent's participation in psycho-social interventions and give them a better understanding of and sense of control over their mental health (Tollefsen et al., 2020b). Adolescents who set personal goals in counseling, showed improved Locus of Control scores with less attribution to external factors and less attribution of mental health to random factors such as luck (Tollefsen et al., 2020b).

Working with goals is a central element in EMOTION, and an important function of the MittEcho app. Focusing on goals can be one way to ensure adaptation and behavioral change. Children enter their goal into the app and evaluate the progress weekly. Though children in the ECHO study are not presented with their own MFS data, answering questions and evaluating goals can trigger reflection on current state and goal progress (Solstad et al., 2019). This again can motivate the child to take own action for improvement, which could improve coping skills. Children are also reminded of own goals by opening the app, and thereby facilitating goal achievement outside group sessions. The weekly update from MFS on each child's goal, may make the group leaders pay more attention to each child's progress, and to a larger degree support and help with goal attainment, contributing to enhanced outcome.

#### Child Involvement

Involvement of youth is hypothesized to facilitate autonomy and coping skills and could mediate the effect of MFS on outcomes (Tollefsen et al., 2020b). Involvement and facilitation of autonomy can also be one way of giving young people more sense of power over their own improvement (Solstad et al., 2019). Though Tollefsen et al. (2020b) did not find an effect of MFS on user involvement, interviews with counselors of first line services for young people indicated that user involvement was a possible factor (Tollefsen et al., 2020a). MFS seems to effect attribution style and locus of control (Tollefsen et al., 2020b), and these may be connected to involvement. MFS can also give participants a different way of being involved and getting a voice. For example, Solstad et al. (2019) found that MFS allowed clients to express themselves without speaking, which for some can be less straining.

#### Collaborative Relation

A recent synthesis of both adult and young mental health patients' experiences of using MFS proposes two meta-themes: patient empowerment and developing collaborative practice (Solstad et al., 2019). Collaborative practice is closely related to the term therapeutic alliance, which has been associated with beneficial outcomes of individual therapy for adults (Miller et al., 2010; Flückiger et al., 2018). A comprehensive review on the effects of collaborative relation among children and youth by Karver et al. (2006) found therapeutic relation to have moderate to strong effect sizes in relation to outcome of therapy. Gondek et al. (2016) did not succeed in finding significant effect on therapeutic alliance when using MFS but notes that the evidence is uncertain. Having a trusting, collaborative relation with group leaders may be equally important in the current context where children attend a group-based intervention.

## Discussion

Our central theme is the optimalization of the EMOTION intervention. In a previous RCT (Martinsen et al., 2019) the EMOTION intervention was shown to be effective. The results suggested that, as an entire program, it was significantly effective, but group leaders, service providers and others reported that it was time-consuming and difficult to prioritize in a busy work schedule. Hence, investigating which of the components in the intervention contribute to positive change, adapting it to fit the needs and restraints among service providers without compromising the benefits is the primary aim of the ECHO study.

The ECHO study uses a factorial design including three components and eight experimental conditions. The multifactorial design allows for the testing of interactions as well as main effects of the components, due to equal distribution of all components within each main effect (Collins et al., 2014). In other words, the design of the study provides an opportunity to optimize the intervention through testing which component or combination of components that are necessary to produce the wanted effect but requires as little effort, time and investment as possible from service providers. What is learned will make implementation easier, and possibly increase sustainability and cost-effectiveness. For instance, we hypothesized that the optimal version of the intervention is the condition using DIGGI, with low parental involvement while group leaders use MFS. In this condition, the intervention is partly digitalised and sufficient parental involvement may be achieved through a brochure with information and guidance. This combination will require less time spent on each group of children by service providers, while (hopefully) retaining the effect of the original longer EMOTION intervention. Using MFS helps providers tailor the intervention, increases involvement of the child and collaboration between the child and providers, increasing the effect of the intervention. All in all this reduces investment in each group and saves time for providers but most importantly, if this proves to be the most optimized condition, the consequences in the long run could be that service providers have time to run more groups and hence help more children, preventing development of emotional problems.

For two of the three components in the ECHO study the hypothesis is that there will be no difference in outcomes between the two levels of the component. In component 1 the EMOTION intervention vs. DIGGI no difference between the levels is suggested because DIGGI, as well as EMOTION, is designed to influence emotion regulation, behavioral patterns, maladaptive thinking, and attitudes in children, regardless of format. This has not yet been tested, and it is possible that although both levels of the component are aimed at the same mediators and mechanisms they will result in different outcomes because of other influences. For instance, the EMOTION intervention might prove to be superior as the children in that level receives more attention from group leader, receive more help to understand and prioritize important aspects of the intervention, and are given better opportunities to see and learn from peers. Few studies have reported details of compliance and completion of internet-based sessions in guided interventions for children (Rooksby et al., 2015). The reported compliance rates in these studies have been somewhat mixed, and the definition of compliance have also varied across studies, making it difficult to infer the role of compliance in treatment effectiveness. In addition, both age and family support may be related to the number of digital sessions completed (Spence et al., 2019).

The same logic holds true for parental involvement. Because the levels (high vs. low involvement) are designed to influence the same mediators, and because results of parental involvement in prevention of child emotional problems are mixed, the hypothesis states that there will be no difference between the two. However, because the high parental involvement level potentially increases parent's understanding through guidance from a group leader and discussing perspectives of other parents. The result might be increased motivation to practice and adhere to different intervention components resulting in better outcomes for the child in the high parental involvement condition.

For MFS the study has hypothesized that using MFS will result in better outcomes than not using MFS. Use of feedback systems in intervention research is relatively new and knowledge about effects of MFS in prevention of childhood emotional problems are scarce and uncertain. However, studies indicate an association between MFS and young people's experience of control and involvement in their own process of change. It has also been associated with increased co-operation between therapist and young people and tailoring of the intervention to the individual participant. Although evidence is uncertain, and mostly related to older age groups than the participants in the Echo study, the hypothesis suggests improved outcomes as a result of using the system. The study will anyway provide initial evidence on whether MFS could work in child prevention.

The conceptual model describes the 3 components of the study, what mediators of change they are aimed at and which mechanisms they might affect to produce a reduction in symptoms of emotional problems in children aged 8–12. Some of the suggested mediators have been proposed as mediators in previous research. Others were chosen based on a documented association between the mediator and emotional problems in children (Kazdin, 2007). This also means that there are other mediators associated with treatment and prevention of emotional problems in children that are not included in the present model (e.g., medication). These were left out as the conceptual model was created based on the EMOTION manual and research on CBT for young people in general.

By understanding the processes that underly change we may be able to optimize our interventions, improving components that work and removing those that do not. Identifying the relation between the intervention, an intervening variable (the mediator) and the outcome provides knowledge about how the intervention works and what needs to be prioritized to retain or enhance effect. In the present study, tests of mediation will be performed and will inform the degree to which the component is effective. The next step would then be to remove components that do not have intended effect, improve those that do, and test this in a new RCT.

The high number of participants in the current effectiveness study (planned *N* = 796), the study setting (including urban and rural schools with group leaders from primary municipal health services), the inclusion and selection process, and the low drop-out rates are all strengths of the study. Together with the factorial design, this study may produce results that enhance our knowledge about mechanisms and mediators of young people's emotional problems and how to better prevent these problems. However, the study also has weaknesses. The ECHO study is conducted during the Covid-19 pandemic, and it is uncertain how this may influence the results. Furthermore, some of the requirements of a formal mediator can be hard to demonstrate in the analyses. Finally, not all mediators proposed in this study can be measured directly without placing a large response burden on participants and is therefore only measured indirectly. The study is executed in a non-clinical context and participants might have limited motivation for answering questionnaires, hence the limited number of measures.

## Conclusion

The conceptual model is the base of the ECHO study. The full factorial design, inspired by a multiphase optimization strategy (Collins, 2018) gives an opportunity to test which component or combination of components provides the best balance between outcome and key constraints as need for efficiency and economy. The factorial design in ECHO can provide knowledge about the effect of different components and therefore add to our knowledge taking us one step further than a traditional RCT does. This is important as it will provide evidence for the effect of different components and guide new research testing interventions based only on effective components, removing those that do not add to effect or altering them to test individually for effect. The results will also have implications for practice, as knowledge of how and why mediators work will give us a chance to optimize each component of the intervention. Together this will retain or improve the quality of the evidence-based intervention EMOTION, save service provider's time and money, while improving access to evidence-based practice for children with emotional problems.

## Data Availability Statement

The original contributions presented in the study are included in the article/supplementary material, further inquiries can be directed to the corresponding author/s.

## Author Contributions

JI: writing original draft, review, and editing. KM, FA, AS, KY, EB, CL, L-MR, IH, JP, LC, PK, and SN: writing, review and editing. All authors contributed to the article and approved the submitted version.

## Conflict of Interest

PK receives royalties from the sales of materials related to the treatment of anxiety in youth. The remaining authors declare that the research was conducted in the absence of any commercial or financial relationships that could be construed as a potential conflict of interest.
